# Structure of *Arabidopsis thaliana *5-methylthioribose kinase reveals a more occluded active site than its bacterial homolog

**DOI:** 10.1186/1472-6807-7-70

**Published:** 2007-10-25

**Authors:** Shao-Yang Ku, Kenneth A Cornell, P Lynne Howell

**Affiliations:** 1 Program in Molecular Structure and Function, Research Institute, Hospital for Sick Children, 555 University Avenue, Toronto, Ontario, M5G 1X8, CANADA; 2 Department of Biochemistry, Faculty of Medicine, University of Toronto, Medical Science Building, Toronto, Ontario, M5S 1A8, CANADA; 3 Department of Chemistry and Biochemistry, Boise State University, 1910 University Drive, Boise, Idaho 83725-1520, USA

## Abstract

**Background:**

Metabolic variations exist between the methionine salvage pathway of humans and a number of plants and microbial pathogens. 5-Methylthioribose (MTR) kinase is a key enzyme required for methionine salvage in plants and many bacteria. The absence of a mammalian homolog suggests that MTR kinase is a good target for the design of specific herbicides or antibiotics.

**Results:**

The structure of *Arabidopsis thaliana *MTR kinase co-crystallized with ATPγS and MTR has been determined at 1.9 Å resolution. The structure is similar to *B. subtilis *MTR kinase and has the same protein kinase fold observed in other evolutionarily related protein kinase-like phosphotransferases. The active site is comparable between the two enzymes with the DXE-motif coordinating the nucleotide-Mg, the D238 of the HGD catalytic loop polarizing the MTR O1 oxygen, and the RR-motif interacting with the substrate MTR. Unlike its bacterial homolog, however, the Gly-rich loop (G-loop) of *A. thaliana *MTR kinase has an extended conformation, which shields most of the active site from solvent, a feature that resembles eukaryotic protein kinases more than the bacterial enzyme. The G- and W-loops of *A. thaliana *and *B. subtilis *MTR kinase adopt different conformations despite high sequence similarity. The ATPγS analog was hydrolyzed during the co-crystallization procedure, resulting in ADP in the active site. This suggests that the *A. thaliana *enzyme, like its bacterial homolog, may have significant ATPase activity in the absence of MTR.

**Conclusion:**

The structure of *A. thaliana *MTR kinase provides a template for structure-based design of agrochemicals, particularly herbicides whose effectiveness could be regulated by nutrient levels. Features of the MTR binding site offer an opportunity for a simple organic salt of an MTR analog to specifically inhibit MTR kinase.

## Background

S-adenosyl-L-methionine (SAM) is an important metabolite in plants as it not only provides the methyl group required for SAM-dependent biomethylation reactions, but also acts as the precursor for the biosynthesis of polyamines, nicotianamine, phytosiderophores, and the plant hormone ethylene [1]. SAM is metabolized to S-adenosylhomocysteine (SAH) in biological methylation, and to 5'-methylthioadenosine (MTA) in many other essential biological processes. While SAH is the product and a potent inhibitor of all SAM-dependent methyltransferases [2], MTA inhibits spermine synthase [3] in polyamine biosynthesis, and 1-aminocyclopropane-1-carboxylic acid synthase [4] in ethylene biosynthesis. Both polyamine and ethylene are important in plant development, growth, stress response and survival [5-7]. To promptly remove the toxic by-product MTA and to ensure sufficient supply of methionine and SAM, a methionine salvage pathway that recycles the methylthio moiety of MTA to methionine has evolved in almost all organisms. In plants, this pathway is also known as the Yang cycle [8]. In this cycle MTA is first depurinated to 5-methylthioribose (MTR), and then phosphorylated to form MTR 1-phosphate (MTR 1-P). MTR 1-P then undergoes isomerization, dehydration, enolization and dephosphorylation to form the acireductone, 1,2-dihydroxyl-3-keto-5-methylthiopentene. This acireductone is subsequently converted to 2-keto-4-methylthiobutyrate and then transaminated to methionine [8,9].

Metabolic variation in how MTA is recycled to methionine exists between different species. In bacteria and plant cells, two enzymes, MTA nucleosidase and MTR kinase, are required to convert MTA to MTR-1-P while in mammalian cells, MTA phosphorylase [10] phosphorylizes MTA to MTR 1-P in a single step. This metabolic difference has been explored and analogs of MTA and MTR have been synthesized for the development of specific antibiotics and herbicides [11-14]. MTR kinase is not an essential enzyme for the organisms' survival if methionine is present in the growth medium [15], but in the absence of methionine, expression of MTR kinase will prompt an organism to uptake MTA and/or MTR from the environment to replenish its sulfur pool [15,16]. Interestingly, even when sulfur is not depleted in the environment, numerous analogs of MTR have demonstrated selective growth inhibition of MTR kinase-containing bacteria [11,12]. Not surprisingly, the bactericidal activity of these MTR analogs, particularly trifluoromethylthioribose, is enhanced if the *de novo *methionine synthesis pathway is also blocked [17]. In plants, the expression of MTR kinase is also up-regulated under sulfur-limiting conditions, although the expression is not correlated with ethylene biosynthesis [15,16,18]. While most studies of MTR analogs have been performed on bacterial MTR kinases for the design of novel antibiotics, similar principles can be applied to the plant system to develop new herbicide and/or herbicide-resistant transgenic crops that would improve agriculture efficiency.

The modern rational approach to new agrochemical discovery includes structure-based design approaches [19], which requires a detailed understanding of the target enzyme's structure, catalytic mechanism and substrate specificity. To this end, we present the first structural analysis of *A. thaliana *MTR kinase in complex with ADP and MTR.

## Results and Discussion

### Overall fold of MTR kinase

**Figure 1:**
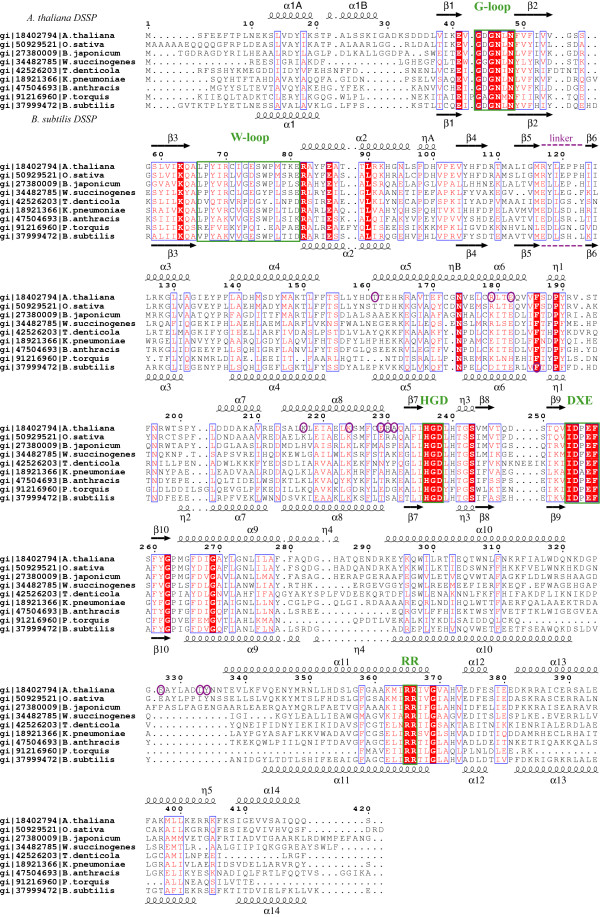
Multiple sequence alignment of selected MTR kinase sequences mapping the secondary structural elements found in *A. thaliana *(top) and *B. subtilis *(bottom) MTR kinase structures. The protein sequences aligned are from *Arabidopsis thaliana*, *Oryza sativa*, *Bradyrhizobium japonicum*, *Wolinella succinogenes*, *Treponema denticola*, *Klebsiella pneumoniae*, *Bacillus anthracis*, *Psychroflexus torquis *and *Bacillus subtilis*. Their gene identifiers (gi) from the National Center for Biotechnology Information (NCBI) are also listed. The secondary structural elements were defined according to *DSSP *[48], numbered by the order of their appearance, and named to be consistent between the two enzymes. α helices are presented as curly lines and β strands by arrows. η stands for a 3_10 _helix. According to *DSSP*, the *A. thaliana *enzyme does not have the η2 helix found in the *B. subtilis *enzyme. The multiple sequence alignment was performed using the program *T-Coffee *[49], and the figure prepared using *ESPript *[50]. Strictly conserved residues are denoted in white and framed in blue boxes with a red background; residues conserved in at least 70% of the sequences are denoted in red and framed in blue boxes with a white background. The G-loop, the W-loop, the Mg-binding DXE-motif, the HGD catalytic loop, and the MTR-binding RR-motif are framed in green boxes. Residues circled in purple are involved in dimer formation. The linker region connecting the N-lobe and C-lobe is also indicated.

**Figure 2:**
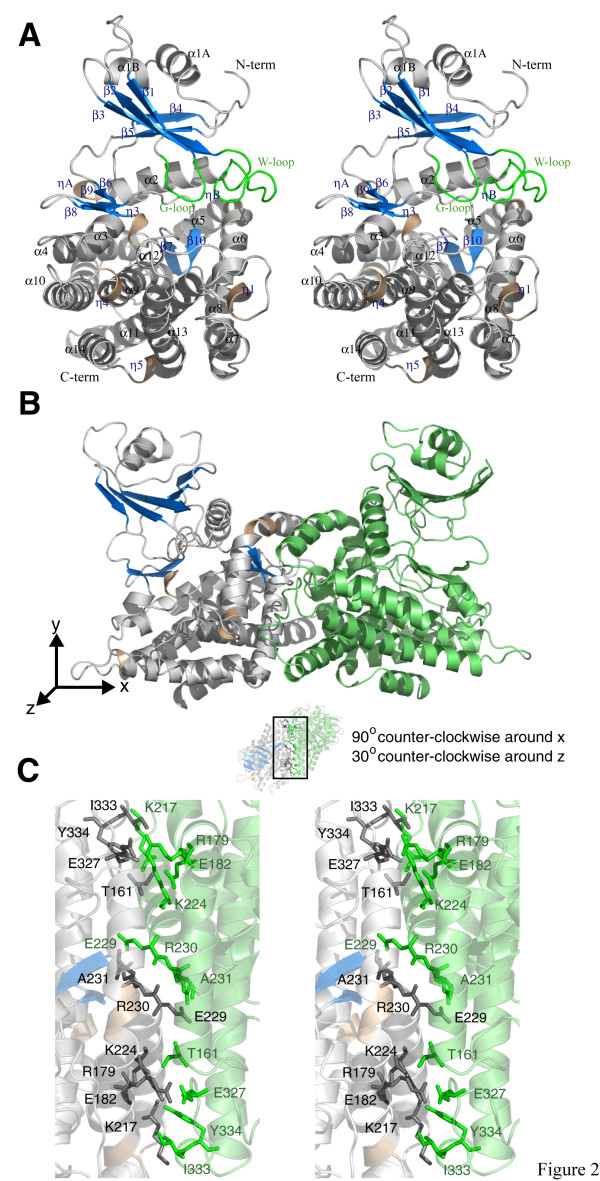
Structure of *A. thaliana *MTR kinase. (a) Stereo representation of the plant MTR kinase monomer. Monomer A of the MTRK-ADP-MTR complex is shown, with the nucleotide and substrate omitted. α helices are represented as grey coils with the 3_10 _helices as wheat coils. β strands are represented as blue arrows and loops as grey tubes. The G-loop and W-loop are coloured in green. (b) Dimeric structure of the complex with monomer A coloured as in (a) and monomer B in green. (c) Stereo representation of detailed interactions between the two monomers. Figure 2 was prepared using *PyMOL *[51].

The structure of *Arabidopsis thaliana *MTR kinase has two lobes: a smaller N-lobe and a larger C-lobe connected by a linker region (residues 117–123) (Fig. 1 and 2a). The N-lobe is composed of five anti-parallel β strands (β1–β5) flanked by three short α helices (α1A, α2B and α2) in an ααβββαββ topology (Fig. 2a). Helices α1A and α1B are located at the N-terminus, while helix α2 is inserted between strands β3 and β4 and lies beneath the β strands near the C-lobe (Fig. 2a). The larger C-lobe is predominantly α helical with twelve α helices (α3–14) and six short 3_10 _helices (ηA-B, η1 and η3–5) along with four short β strands (β7–β10) between helices α8 and α9, and a short strand, β6, at the C-terminal end of the linker region. Strand β8 forms an anti-parallel β turn with strand β9, while strands β7 and β10 form a second anti-parallel sheet. The bilobal structural organization as well as the topology profile of MTR kinase is consistent with that found in eukaryotic protein kinases.

**Figure 3:**
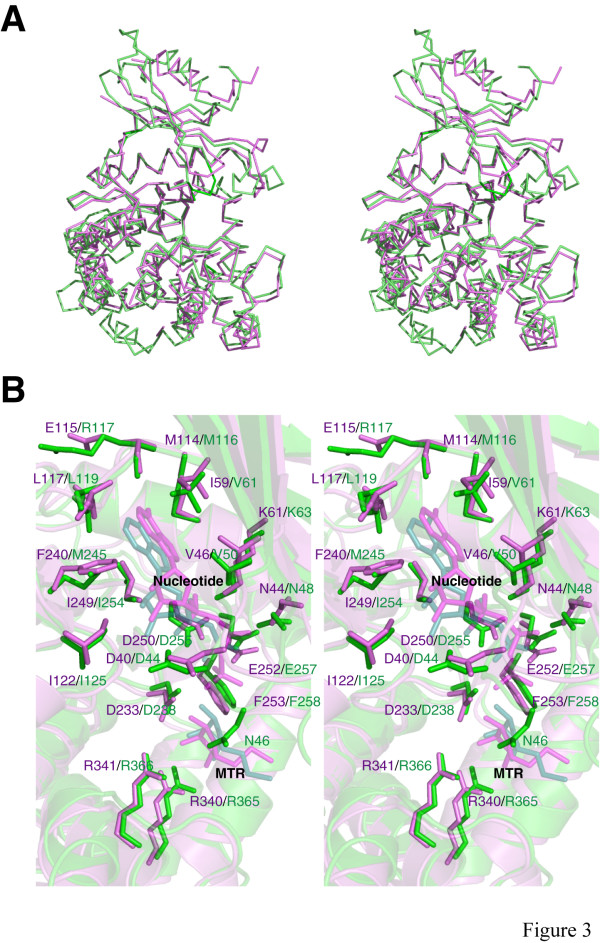
(a) Stereo representation of C^α ^superimposition of *B. subtilis *MTR kinase AMPPCP-MTR complex (PDB: 2PUN) in violet and *A. thaliana *MTR kinase ADP-MTR complex (PDB: 2PYW) in green. (b) Stereo stick presentation of the active sites of the *B. subtilis *MTR kinase in violet and *A. thaliana *MTR kinase in green. The two structures have been superimposed as in (a) and are shown in transparent cartoon. Monomer A is shown. The nucleotides and substrate MTR are shown in transparent stick representation (and labelled) in order to show the residues behind. This figure was prepared using *PyMOL *[51].

*A. thaliana *MTR kinase has 35% amino acid sequence identity with *Bacillus subtilis *MTR kinase. The two enzymes share a similar secondary structure profile (Fig. 1) [20] and have a C^α ^root mean square deviation (RMSD) of 1.7 Å (Fig. 3a). MTR kinase is structurally similar to other evolutionarily related protein kinase-like phosphotransferases. A comparison of the structure of *A. thaliana *MTR kinase to other known structures using *Dali *reveals that the enzyme resembles choline kinase (PDB:1NW1) [21], APH Type IIIa (APH(3')-IIIa) (PDB:1J7I) [22], and the archaeal atypical serine protein kinase Rio2 (PDB:1ZAR) [23] with Z-scores of 14.4, 12.5 and 11, C^α ^RMSD's of 4.8 Å, 4.6 Å and 3.6 Å, and aligned sequence identities of 14%, 13% and 19%, respectively [20].

### Quaternary structure

The structure of *A. thaliana *MTR kinase has two subunits in the asymmetric unit related by a non-crystallographic 2-fold (Fig. 2b). Approximately 1300 Å^2 ^or 6.7% of the surface area of each monomer is buried at the dimer interface [24]. Even though the plant enzyme underwent a complex multi-step refolding process [25], its dimeric structure resembles that of the bacterial enzyme [26]. Size exclusion chromatography of *A. thaliana *MTR kinase (data not shown), the physical characterization of other plant MTR kinases [27] as well as the apparent cooperative kinetics observed for the rice enzyme [15] all suggest that MTR kinase functions as a homodimer. The inter-subunit interactions in the dimer interface of *A. thaliana *MTR kinase are predominantly symmetrical and are concentrated at the C-terminus of helix α8 (Residues K217, K224, E229, R230 and A231). Additional inter-subunit interactions are found at the N-terminus of helix α5 (T161), the C-terminus of helix α6 (R179 and E182), as well as the loop between helices α10 and α11 (E327, I333 and Y334) (Fig. 1). The interactions between these residues are depicted in Fig. 2c. Interestingly, when comparing the dimeric interfaces of the *B. subtilis *and *A. thaliana *enzymes, the identities of the residues involved in dimerization are not conserved (Fig. 1). The interactions are nevertheless concentrated in approximately the same region of the protein, especially the C-terminal end of helix α8, and result in exactly the same dimeric structure.

Although the tertiary structures of MTR kinase and other protein kinase-like phosphotransferases are highly similar, their quaternary structures are distinct. While MTR kinase dimerizes mainly *via *interactions between helices α8, choline kinase dimerizes *via *interactions between helices that are structurally equivalent to helices α2 of MTR kinase. Although crystal structures of APH show the protein as dimers [28,29], they are not physiologically relevant as the enzyme is observed as a monomer in solution [30]. Rio2 protein kinase is also observed as a monomer [23].

### Nucleotide binding site

**Figure 4:**
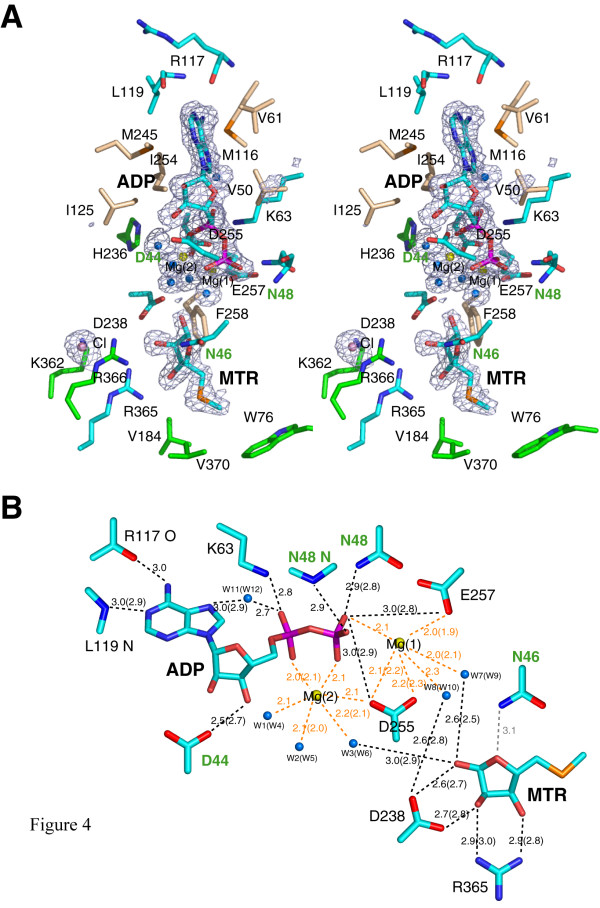
The active site of *A. thaliana *MTR kinase. (a) Stereo representation of the active site. The product ADP and the substrate MTR and nearby interacting residues are drawn in stick representation with C, N, O, P and S atoms coloured in cyan, blue, red, magenta, and orange, respectively. Hydrophobic residues are shown with their C atoms coloured in wheat. Residues that do not directly interact with ADP or MTR but are discussed in the text are shown with their C atoms coloured in green. The magnesium ions, chloride ion and water molecules are shown as yellow, violet and blue spheres, respectively. The σ_A_-weighted *Fo*-*Fc *ligand (and water) omit map in blue mesh was calculated using *CCP4 *[52] and is contoured at 3σ. (b) Schematic diagram showing the hydrophilic interactions less than 3 Å between the ligands and the protein. The colour schemes of the atoms are as described in Panel (a). The distances in Å between the ligands and the protein are for monomer A while those for monomer B are in parentheses. If the two distances are the same, only one value is shown. The distances around the two octahedrally coordinated Mg(II) ions are labelled in orange. Residues from the G-loop are labelled in green. The distance between the N^δ ^amide nitrogen of N46 in the G-loop and the O4 of MTR is greater than 3 Å and hence is shown in grey instead of black. Both panels were prepared using *PyMOL *[51].

The nucleotide binds in the cleft region between the two lobes of the protein. The adenine ring of the nucleotide binds near the linker region in a hydrophobic pocket. The N6 and N1 nitrogens of the adenine ring are hydrogen-bonded to the backbone carbonyl oxygen of R117 and the amide nitrogen of L119, respectively (Fig. 4). The strictly conserved K63 also interacts with the α-phosphoryl group of the nucleotide *via *an ionic interaction. A structurally conserved water molecule (W11 in monomer A; W12 in monomer B) donates two hydrogen bonds to the nucleotide: one to the N7 nitrogen of the adenine ring and the other to the O1 oxygen of the nucleotide's α-phosphoryl group (Fig. 4). This water molecule is also observed in all of the bacterial MTR kinase nucleotide complex structures determined to date [26].

### Functionally important loops

There are four conserved loop regions in MTR kinases: the G-loop between strands β1 and β2, the HGD catalytic loop between strand β7 and the 3_10 _helix η3, the Mg(II) binding DXE-motif between strands β9 and β10, and the semi-conserved W-loop between strand β3 and helix α2 (Fig. 1). The G-loop of MTR kinases has a highly conserved GXGNXN motif (residues 43–48) and is structurally analogous the "nucleotide positioning loop" in APH(3')-IIIa [31] and the "Gly triad" GXGXXG motif found in many eukaryotic protein kinases.

### Gly-rich loop (G-loop) and solvent accessibility

**Figure 5:**
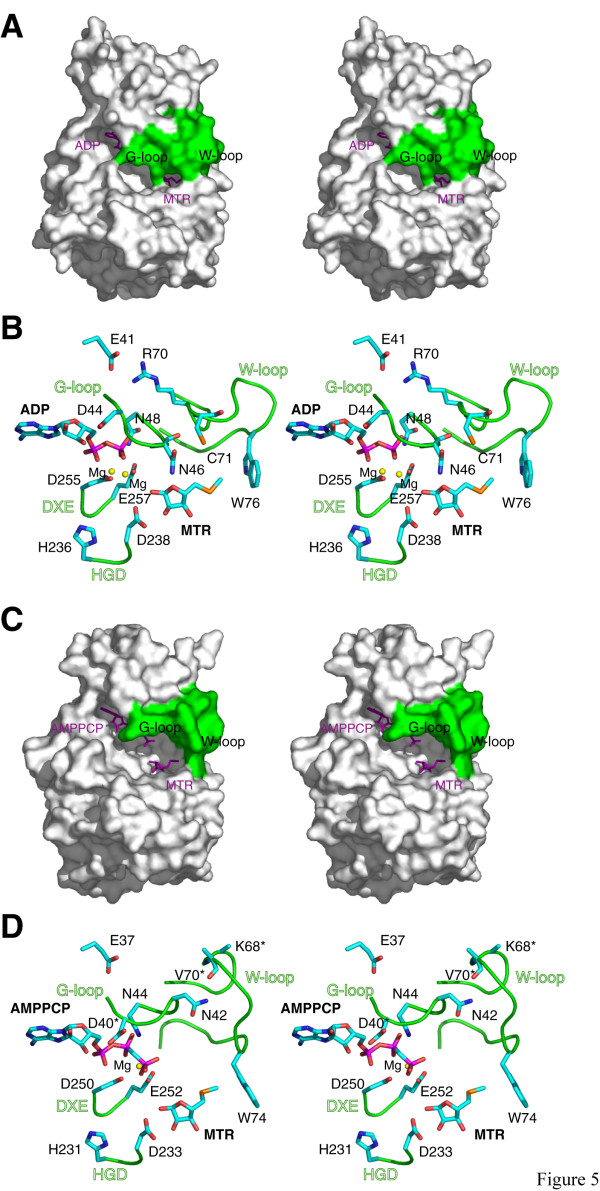
Comparison of the nucleotide binding pockets and loop conformations. Stereo surface representation of the *A. thaliana *MTR kinase ADP-MTR complex and *B. subtilis *MTR kinase AMPPCP-MTR complex are shown in panel (a) and (c), respectively with the G- and W-loop coloured in green and the ligands shown as purple sticks. Stereo cartoon representations of the four functionally important loops, the G-loop, the W-loop, the Mg-binding DXE-motif, and the HGD catalytic loop, found in the *A. thaliana *and *B. subtilis *enzymes are shown in (b) and (d), respectively. Substrates and important residues discussed in the text are shown in stick presentation with the same colour scheme as in Figure 4b. Residues labelled with an asterisk indicate that disordered side chains are observed in at least one subunit of all known structures of the *B. subtilis *enzyme. This figure was prepared using *PyMOL *[51].

In *A. thaliana *MTR kinase, the G-loop plays a similar role in anchoring and coordinating the bound nucleotide. Residue D44 of the G-loop, although not absolutely conserved, makes a ~2.6 Å hydrogen bond to the nucleotide's O3' ribosyl oxygen, while the backbone and terminal amide nitrogen's of N48 interact with the β-phosphoryl oxygens. The G-loop of *A. thaliana *MTR kinase has an extended conformation, with interactions formed between N46 and the O4 oxygen of MTR (Fig. 4b and 5a). This extended G-loop conformation is different from that observed in the bacterial MTR kinase, as the equivalent residue in the *B. subtilis *enzyme, N42, is over 10 Å away from the O4 oxygen of MTR (Fig. 5d). In most of the bacterial structures determined to date [26], the G-loop is disordered, and in no case does it interact with MTR. A consequence of this extended G-loop in the *Arabidopsis *enzyme is that it shields the active site from solvent (Fig. 5a), a feature that more closely resembles a eukaryotic protein kinase active site than that of the *B. subtilis *MTR kinase (Fig. 5c). Since the G-loop appears to deny the entry and/or exit of nucleotides, conformational changes – loop movement at a minimum – will need to occur to allow nucleotide entry to the *apo*-form the enzyme.

### DXE-motif and Mg binding

In the *A. thaliana *MTR kinase structure, two Mg(II) ions assist in the binding of the ADP. Mg(II) ions are important for MTR kinase activity. The kinase activity is optimal in the presence of Mg(II) ions, attenuated to 20% in the presence of Mn(II) ions, and abolished in the presence of Ca(II) ions [32]. Residues D255 and E257 are part of the strictly conserved DXE-motif on the loop between strands β9 and β10 and are essential for Mg(II) binding. The first magnesium ion, Mg(1), is chelated by the carboxyl O^δ ^oxygens of D255, the carboxyl O^ε ^oxygen of E257, the O2 oxygen of the β-phosphoryl group of ADP, and two water molecules. These water molecules in turn interact with the O1 oxygen of MTR and the catalytic residue D238 (see below) (Fig. 4b). The second magnesium ion, Mg(2), is chelated by the α- and β-phosphoryl oxygens of ADP, the O^δ ^oxygen of D255 and three water molecules. One of the water molecules that interacts with Mg(2) (W3 in monomer A and W6 in monomer B) also interacts with O1 oxygen of MTR (Fig. 4b). Unlike the bacterial MTR kinase where the water molecules surrounding the Mg(II) ions have poor electron density, both Mg(II) ions in the *A. thaliana *ADP-enzyme complex are coordinated in the most energetically favorable octahedral geometry.

Examination of amino acid sequences reveals that for all MTR kinases, except those from *Nocardioides*, *Streptomyces *and *Clostridium *species, the DXE-motif can be extended to a longer IDXEF motif (Fig. 1). The DXE-motif is always followed by a phenylalanine, F258 in *A. thaliana *MTR kinase, which is involved in MTR binding (see below). The significance of the DXE-motif is that this consensus sequence is analogous to the well defined DFG-motif that marks the start of the "activation loop" in protein kinases [33]. Unlike other phosphotransferases such as APH(3')-IIIa or cAMP-dependent protein kinase (PKA), which use the aspartate in the DFG-motif (e.g. D208 in APH(3')-IIIa and D184 in PKA) and a second asparagine (e.g. N195 in APH(3')-IIIa and N171 in PKA) from the C-lobe to chelate the divalent metal ions, MTR kinase uses both acidic residues in the DXE-motif to bind Mg(II).

### RR-motif in the MTR binding site

The substrate MTR is located beneath the nucleotide in the active site. In *A. thaliana *MTR kinase, MTR is bound in the higher energy α-ribofuranose anomer with the substrate's O1 hydroxyl *cis *to the O2 and O3 hydroxyl groups (Fig. 4a). MTR is also observed in the same α-anomer in the bacterial enzyme-substrate complex [26] (Fig. 3b). In the plant enzyme, the α-conformation is stabilized by interactions with D238 and R365 as well as F258, which interacts with the substrates hydrophobic face (Fig. 4b). The O1 and O2 oxygens of MTR form hydrogen bonds (~2.7 Å) with the carboxyl oxygens of D238 of the strictly conserved HGD-motif; while the O2 and O3 oxygens of MTR forms heteronuclear hydrogen bonds (~2.9 Å) with the guanidium nitrogens of R365 of the strictly conserved twin arginine RR-motif (Fig. 1). The second arginine R366 does not interact directly with MTR but may facilitate the release of the anionic product, MTR 1-P. In the *A. thaliana *enzyme, a lysine (K362) is located near the RR-motif (Fig. 4a), and to mitigate the electrostatic repulsion between these three positively charged residues, a chloride ion is found interacting with K362.

Interestingly, examination of the multiple sequence alignment (Fig. 1) shows that the residue equivalent to K362 in the *A. thaliana *enzyme, three residues upstream of the RR-motif, is always either a lysine or a glutamate residue in all known sequences of MTR kinases. Most bacterial MTR kinases including the well-studied *Klebsiella pneumoniae *and all of the *Bacillus *enzymes, have a glutamate at this position. This glutamate will help neutralize the positive charge imposed by the RR-motif in the MTR binding site. A lysine residue near the RR-motif, as found in the plant *A. thaliana*, *Oryza sativa *(rice), *Vitis vinifera *(grape), and nitrogen-fixing bacteria *Bradyrhizobium japonicum *and *Wolinella succinogenes*, would perturb MTR binding by charge repulsion when the pH falls below the pKa of lysine. Thus, the K362 in *A. thaliana *MTR kinase could potentially explain why this enzyme's *in vitro *activity is optimal at pH 9, near the pK_a _of lysine, while the optimal activity for *K. pneumoniae *MTR kinase occurs at pH 7 (Reference [34] and see Additional file 1).

### W-loop and MTR binding specificity

The W-loop between strand β3 and helix α2, named after the semi-conserved tryptophan (W) at the tip of this loop, appears to modulate the size of chemical group at the 5-position that can fit into the MTR binding site. In *A. thaliana *MTR kinase, the 5-methylthio moiety of the MTR appears to be capped by W76 of the W-loop and residues V184 and V370 from the C-lobe (Fig. 4a). These three residues would appear to restrict the length of the alkylthio moiety at the 5-position. Although these three residues are not strictly conserved across MTR kinase sequences, they are structurally and functionally equivalent to residues W74 of the W-loop, and L180 and L345 in *B. subtilis *MTR kinase. However, many MTR analogs with bulky extensions at the 5-position have been shown to be substrates and/or inhibitors of MTR kinase [11,35], although the bulkiness of the substitution does not correlate with their inhibitory concentrations [36]. In fact, these inhibitors have comparable K_m _values (in the μM range) to the native substrate in many bacterial and plant MTR kinases [15,32,33]. Moreover, comparison between *A. thaliana *and *B. subtilis *MTR kinase shows that both the 5-methylthio moiety of MTR and the W-loop adopt different conformations in the otherwise comparable overall active sites (Fig. 3b and 5). These results suggest that the W-loop needs to be flexible to allow the entry of different sized MTR analogs to the active site. This hypothesis is consistent with the observation in *B. subtilis *MTR kinase structures that the density of the W-loop is often disordered [26], implying its flexibility.

### Conformational variations

In the structural alignment of bacterial and plant MTR kinase, the bound ADP in *A. thaliana *is shifted toward the C-lobe when compared to the nucleotide in the *B. subtilis *enzyme (Fig. 3b). This observation raises the question whether the plant MTR kinase has a more closed conformation between the two-lobes than its bacterial homolog. Although the overall structural alignment of the two enzymes shows no major conformation changes, a minor rigid body shift of 1–1.5 Å in the N-lobe becomes apparent when only the C-lobes of the two structures are aligned. However, this displacement does not definitively demonstrate a potential inter-lobal movement in MTR kinase on substrate binding, because the intrinsic variation resulting from the sequence variation between the two enzymes – even neglecting the intrinsic structure coordinate errors of both structures – is more than 1.5 Å. In other words, a 1.5 Å displacement observed between the N-lobes is within the expected structural variation of two non-identical protein sequences [37].

Despite the low overall C^α ^RMSD between the structures of *A. thaliana *and *B. subtilis *MTR kinase, the conformations of the G- and W-loop in the two enzymes differ by ~3.8 Å and 5.3 Å in their C^α ^RMSDs, respectively (Fig. 5b and 5d). This observation is striking as the sequence identity of the 6-residue G-loop between the two enzymes is 100%, and that of the 16-residue W loop is 50% (Fig. 1). These RMSD values are close to the expected values if two random loops of the same length are taken from the Protein Data Bank and structurally aligned [38]. The conformational variations between the two enzymes are the result of different interactions outside the loop regions. In the *A. thaliana *enzyme, the highly conserved E41, preceding the G-loop, interacts with R70 of the W-loop *via *an ionic interaction (Fig. 5b). The corresponding E37 and K68 in the *B. subtilis *enzyme do not interact (Fig. 5d). Moreover, in the plant enzyme, the main chain carbonyl oxygen of N46 (G-loop) forms a hydrogen bond to the main chain nitrogen of C71 (W-loop) (Fig. 5b), but in the bacterial enzyme, the corresponding N42 (G-loop) interacts with main chain carbonyl oxygen of V70 (W-loop) *via *the side chain N^δ ^nitrogen instead (Fig. 5d). The difference in loop conformations could also be due to the differences in how they interact with the substrate in the homologous active sites (Fig. 3b). Until an *apo*-form of *A. thaliana *MTR kinase becomes available, it would be difficult to determine whether the variations in the loop conformations are substrate induced.

### ATPase activity in MTR kinase

The *A. thaliana *MTR kinase complex was prepared by co-crystallization with excess but equal amount of ATPγS and MTR, but surprisingly, ADP and MTR were found in the active site. Thus, consistent with the soaking and co-crystallization experiments of bacterial MTRK in the presence of ATP [26], the plant enzyme appears to be able to hydrolyze the weakly hydrolysable ATPγS to ADP. Without extensive kinetic characterization it is hard to know whether this ATP hydrolysis is coupled to phosphorylation of MTR, because while only MTR was found in the active site, it might bind preferentially to MTR 1-P. The observation that *A. thaliana *MTR kinase, like its bacterial homologue, may have considerable ATPase activity in the absence of the substrate MTR will have an impact on the strategies used to design herbicides that target the enzyme.

## Conclusion

The structure of a eukaryotic MTR kinase has been determined for the first time. Although the enzyme has been refolded into its active state, it has comparable tertiary and quaternary structures to its bacterial homolog. The active site is comparable between the two enzymes with the DXE-motif coordinating the nucleotide-Mg, the D238 of the HGD catalytic loop polarizing the MTR O1 oxygen, and the RR-motif interacting with MTR. The G-loop and W-loop of the *A. thaliana *MTR kinase adopt different conformations, a consequence potentially of differences in their microenvironments. Unlike its bacterial homolog, the G-loop of *A. thaliana *MTR kinase has an extended conformation, which shields  most of the active site from solvent, a feature that resembles a eukaryotic protein kinase more closely than the bacterial enzyme. The *A. thaliana *MTR kinase has optimal activity at higher pH than its bacterial homolog and this can be explained by the presence a lysine, rather than a glutamate, near the RR-motif in MTR binding site. The structure of *A. thaliana *MTR kinase also provides a template for structure-based design of agrochemicals – particularly herbicides whose effectiveness could be regulated by nutrient levels. Generic protein kinase inhibitors likely inhibit MTR kinase as they inhibit the remotely homologous APH enzymes [39], but these kinase inhibitors are often associated with complex steps of synthesis and isolation and high cost. The strictly conserved RR-motif in MTR binding site along with the nearby catalytic aspartate offers an opportunity for a simpler organic salt of an MTR analog to specifically inhibit MTR kinase at lower cost.

## Methods

*A. thaliana *MTR kinase was cloned, and expressed in *E. coli*, and purified from inclusion bodies as a linear peptide, refolded into its active form and co-crystallized with substrates as described previously [25]. The effect of pH on MTR kinase activity was examined using a modification of the standard enzyme assay [34] in which the imidazole buffer (pH 6.5) was replaced by 100 mM sodium phosphate (pH 4 – 9.5). Assays used 300 ng of recombinant enzyme and were performed in triplicate. Although prior to co-crystallization the enzyme was incubated with ATPγS and MTR, only ADP and MTR were found in the resulting structure. The crystals belong to space group *C*2 (*a *= 162.5 Å, *b *= 83.2 Å, *c *= 91.1 Å, β = 117.8°) with a dimer per asymmetric unit. Prior to data collection the crystal was soaked in a solution containing 25% (v/v) ethylene glycol, 7.5% (w/v) PEG8000, 75 mM sodium cacodylate pH 6.5, and 0.15 M magnesium acetate for 5 seconds and flash frozen in a nitrogen cryo-stream. Data were collected to 1.9 Å resolution at Station X8C, National Synchrotron Light Source, Brookhaven National Laboratory, and processed using d*TREK [40] and the *DREAR *program package [41].

The structure of *A. thaliana *MTR kinase was determined by molecular replacement using the program *Phaser *[42-44] and the *B. subtilis *enzyme as the search model [45]. Cycles of structure refinement were then alternated between *Refmac *[46] and model building using *Coot *[47]. The final structural model of *A. thaliana *MTR kinase has R/R_free _factors of 16.3%/19.7%. Both monomers in the asymmetric unit have bound ADP and MTR molecules as well as two magnesium and one chloride ions. An additional magnesium ion was found to participate in crystal packing, and a total of five ethylene glycol molecules and 778 water molecules have been modeled in the asymmetric unit. *A. thaliana *MTR kinase has 420 residues. Residues 1–2 and 420 of monomer A, and 1, 30–36 and 420 of monomer B could not be modeled due to poor or missing electron density. The side chains of residues 32, 288–289, and 380 of monomer A, and residues 2–5, 56, 418 of monomer B were also truncated to alanine due to poor electron density. The data, refinement and structure validation statistics are summarized in Table 1.

**Table 1:** Diffraction Data and Crystallographic Refinement Statistics

Space Group	*C2*
Cell dimensions (Å, °)	*a *= 162.5, *b *= 82.2, *c *= 91.1 β = 117.8
Wavelength (Å)	1.0
Resolution (Å)	80.6 – 1.9
Average redundancy^a^	3.4 (2.9)
R_merge_^b ^(%)^a^	4.5 (17.5)
Completeness (%)^a^	98.7 (90.8)
<I>/σI^a^	16.9 (4.8)
Number of reflections (working/test)	78352/4105
Number of protein atoms/heteroatoms^c^	6648/103
Number of water molecules	778
R_cryst_/R_free _(%)^d^	16.3/19.7
RMS deviation from ideal values	
Bond length (Å)	0.014
Bond angle (°)	1.4
Average B factor (Å^2^)	
Proteins	16.4
Ligands (ADP and MTR)	16.4
Magnesium ions	18.0
Chloride ions	20.7
Water molecules	25.3
Ramachandran Plot^e^	
Total Favoured (%)	97.8
Total Allowed (%)	100
DPI^f ^coordinate error based on R_free _(Å)	0.13

^a^Values in parentheses corresponds to the value in highest resolution shell (1.97–1.90Å).

^b^R_merge _= ∑∑|I (k) - <I>|/∑I (k) where I (k) and <I> represent the diffraction intensity values of the individual measurements and the corresponding mean values. The summation is over all unique measurements.

^c^Heteroatoms include ADP, MTR, ethylene glycol, magnesium and chloride ions.

^d^R_cryst _= ∑|F_obs _- F_calc_|/∑|Fobs|; R_free _is R_cryst _for the 5% cross validated test data.

^e^As defined by the Ramachandran plot in *MolProbity *[53].

^f^Cruickshank's diffraction component precision index (DPI) [54] as an estimate of coordinate error.

## List of abbreviations

ADP: adenosine 5'-diphosphate; MTR: 5-methylthioribose; PDB: Protein Data Bank; SAM: S-adenosyl-L-methionine; ATP: adenosine 5'-triphosphate; SAH: S-adenosylhomocysteine; MTA: 5'-methylthioadenosine; MTR 1-P: 5-methylthioribose 1-phosphate; APH(3')-IIIa: 3',5"-aminogylcoside phosphotransferase type IIIa; cAMP: cyclic adenosine monophosphate; PKA: protein kinase A; RMSD: root mean square deviation; ATPγS: adenosine 5'-(β,γ-thio)triphosphate; FAD: flavin adenine dinucleotide; NAD(P)H: nicotinamide adenine dinucleotide (phosphate); AMPPCP: β,γ-methyleneadenosine 5'-triphosphosphate.

## Coordinate

The coordinates of the *A. thaliana *MTR kinase in complex with ADP and MTR have been deposited in the Protein Data Bank, accession number 2PYW.

## Authors' contributions

S-YK – sub-cloning, expression, purification, refolding, crystallization, data collection, structure determination and analysis, and writing the manuscript. KAC – cloning, expression, kinetic analysis, and synthesis of MTR. PLH – concept, analysis, writing and finalizing the manuscript. All authors have read and approved the final manuscript.

## Supplementary Material

Additional file 1 Maximum activity versus pH. Percent maximum activity versus pH for *A. thaliana *(red) and *K. pneumoniae *(blue) MTR kinase. The *A. thaliana *MTR kinase activity was measured as decribed in the Material and Methods, the results for *K. pneumoniae *are from reference [34]. Click here for file
