# Supplementary material for: Structure of Arabidopsis thaliana 5-methylthioribose kinase reveals a more occluded active site than its bacterial homolog
Source: BMC Struct Biol. 2007 Oct 25;7:70. doi: 10.1186/1472-6807-7-70 (PMC2194712; doi:10.1186/1472-6807-7-70)
Supplement: Additional file 1 — Maximum activity versus pH. Percent maximum activity versus pH for A. thaliana (red) and K. pneumoniae (blue) MTR kinase. The A. thaliana MTR kinase activity was measured as decribed in the Material and Methods, the results for K. pneumoniae are from reference [34]. [file 1472-6807-7-70-S1.pdf]

Additional File 1

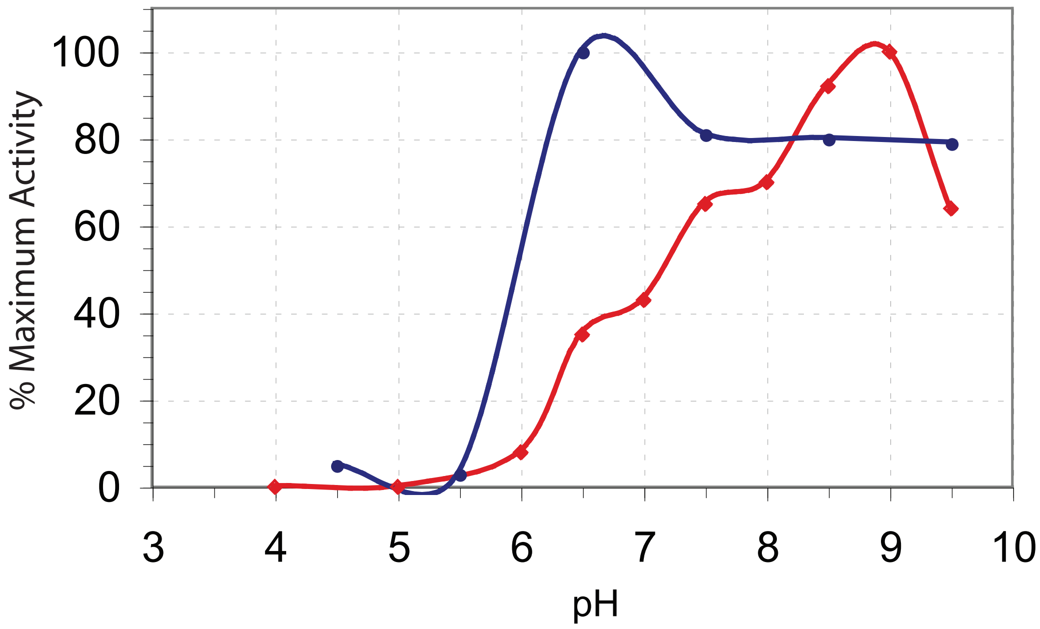

Percent maximum activity versus pH for A. thaliana (red) and K. pneumoniae (blue) MTR kinase. The A. thaliana MTK kinase activity was measured as described in the Material and Methods, the results for the K. pneumoniae are from reference [34] .
